# A Quick Test of cognitive speed is sensitive in detecting early treatment response in Alzheimer's disease

**DOI:** 10.1186/alzrt53

**Published:** 2010-10-15

**Authors:** Sebastian Palmqvist, Lennart Minthon, Carina Wattmo, Elisabet Londos, Oskar Hansson

**Affiliations:** 1 Clinical Memory Research Unit, Department of Clinical Sciences Malmö, Lund University, S-205 02 Malmö, Sweden; 2 Neuropsychiatric Clinic, Skåne University Hospital Malmö, 205 02 Malmö, Sweden

## Abstract

**Introduction:**

There is a great need for quick tests that identify treatment response in Alzheimer's disease (AD) to determine who benefits from the treatment. In this study, A Quick Test of cognitive speed (AQT) was compared with the mini-mental state examination (MMSE) in the evaluation of treatment outcome in AD.

**Methods:**

75 patients with mild to moderate AD at a memory clinic were assessed with AQT and the MMSE at a pretreatment visit, at baseline and after 8 weeks of treatment with cholinesterase inhibitors (ChEI) initiated at baseline. Changes in the mean test scores before and after treatment were compared, as well as the number of treatment responders detected by each test, according to a reliable change index (RCI).

**Results:**

After 8 weeks of treatment, the AQT improvement, expressed as a percentage, was significantly greater than that of the MMSE (*P *= 0.026). According to the RCI, the cut-offs to define a responder were ≥16 seconds improvement on AQT and ≥3 points on the MMSE after 8 weeks. With these cut-offs, both tests falsely classified ≤5% as responders during the pretreatment period. After 8 weeks of treatment, AQT detected significantly more responders than the MMSE (34% compared with 17%; *P *= 0.024). After 6 months of treatment, the 8-week AQT responders still showed a significantly better treatment response than the AQT nonresponders (22.3 seconds in mean difference; *P *< 0.001).

**Conclusions:**

AQT detects twice as many treatment responders as the MMSE. It seems that AQT can, already after 8 weeks, identify the AD patients who will continue to benefit from ChEI treatment.

## Introduction

An estimated population of more than 29 million people worldwide suffered from dementia in 2005 at a cost of US $315 billion [1]. Of all dementia cases, approximately 60% to 70% have Alzheimer's disease (AD) [2,3]. The treatment of AD consists mainly of cholinesterase inhibitors (ChEI), which improve behavior, activities of daily living, and cognitive functions in AD patients [4]. However, not every patient benefits from this treatment. To enhance the drug efficacy and its cost benefits in AD populations, the published guidelines on drug therapy emphasize the importance of identifying those who have responded positively to the treatment [5-7]. Because of the vast number of patients with AD, the evaluation will predominantly have to be conducted in primary care centers, and a simple and quick evaluation test is therefore desirable. The test should also be reliable and sensitive to the specific cognitive changes caused by the treatment.

**Figure 1:**
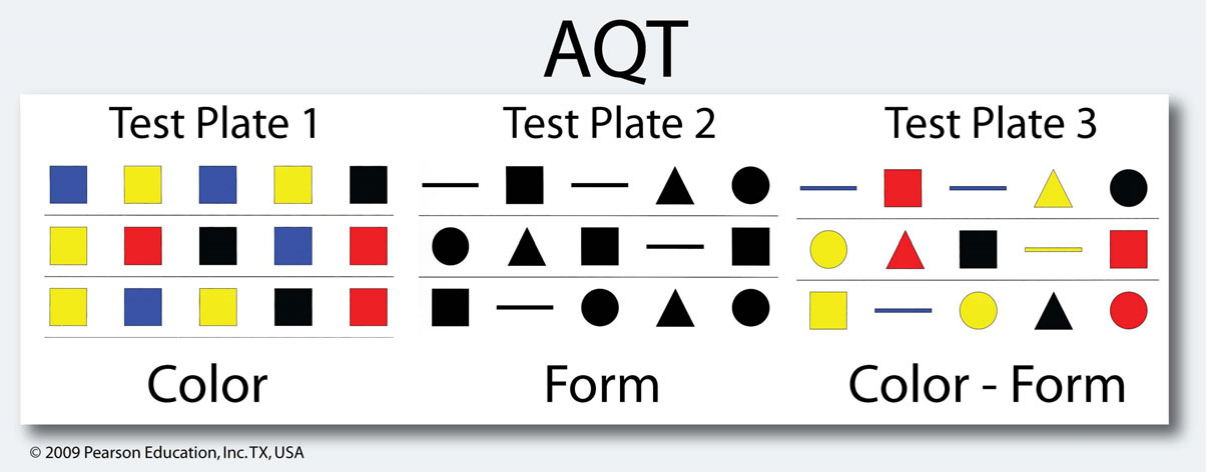
**A sample of AQT**. Each original test plate contains 40 figures. The patient is instructed to quickly name the color of each figure on the first test plate (AQT-C), the form on the second plate (AQT-F), and the color and form on the third plate (AQT-CF) [8]. Only AQT-CF results were analyzed in this study.

A possible candidate for this kind of test is A Quick Test of cognitive speed (AQT), which is a well-validated, sensitive screening tool for cognitive impairment and AD [8] (Figure 1). The AQT takes 3 to 5 minutes to administer, has no ceiling or floor effect, and is independent of gender, education, and culture [9,10]. Previous studies have shown that the AQT activates temporoparietal cortical areas, which are the major brain regions affected in AD [11]. Moreover, one of the main functions measured by the AQT is attention [12], which is the cognitive function that often improves the most from ChEI treatment in AD [13,14]. This makes the AQT a promising test for detecting treatment response in AD. The most common test for evaluating ChEI treatment is the Mini-Mental State Examination (MMSE) [15]. It is also the recommended cognitive test for the evaluation of treatment with ChEI, according to the National Institute for Health and Clinical Excellence (NICE) [5]. This makes MMSE a suitable reference test to compare with the AQT.

An optimal cognitive evaluation test of treatment response will, at a given cut-off, classify very few patients as treatment responders when no treatment is given and as many as possible when treatment is given. To define the cut-off for treatment response, one must consider factors such as cognitive fluctuation of the patients, low test reliability, training effect, and so on. These factors cause changes in test score that are not caused by the treatment and therefore must be accounted for. The most common way of doing that is by establishing a reliable change index (RCI) [16]. RCI is a statistical analysis for detecting individually significant change, and it has been used in more than 500 medical studies.

The aim of this study was to

1. Compare the changes of AQT and the MMSE before and after ChEI treatment in AD patients.

2. Compare the ability of AQT and the MMSE to detect treatment responders according to cut-offs calculated by RCI analyzes.

## Materials and methods

### Patients

The AD patients were enrolled from a part of the Swedish Alzheimer's Treatment Study (SATS) located in the town of Malmö, Sweden [17]. SATS is a prospective, open-label study in routine clinical settings, which have collected patients who have been referred to the Memory Clinic at Malmö University Hospital and have met the criteria for AD according to NINCDS-ADRDA [18]. In addition to a clinical examination by physicians specializing in dementia disorders, the patients were examined with brain computed tomography, routine blood samples, and cerebrospinal fluid analysis. After the baseline visit, treatment with rivastigmine, donepezil, or galantamine was initiated. The patients were followed up in a structured program with assessments at baseline, 8 weeks, 6 months, and semiannually thereafter. For patients to be enrolled from SATS to this study, they had to have MMSE and AQT color-form (AQT-CF) scores from a visit at a predefined time period of 1 to 6 months before baseline, the baseline visit, and the visit at 8 weeks after baseline. The MMSE and AQT scores had to be from the same occasions. Only patients with an MMSE score of 13 points or more and an AQT-CF score of 190 seconds or less at baseline were included because test changes are difficult to assess in patients with very poor test performance, because of low reliability [19]. The cut-offs were predefined and not based on the current study population, which otherwise could introduce selection bias. The inclusion criteria generated a study population of 75 AD patients. They had been followed at the clinic over a mean ± standard deviation (SD) period of 32 ± 19 months and had been reviewed by the study doctors S.P. and O.H. from a longitudinal perspective in regard to diagnosis accuracy. All patients lived at home and had a mean age of 77 ± 6.7 years. Seventy-one percent were women. Sixty-five percent were treated with galantamine, 18% with rivastigmine, and 17% with donepezil. The mean doses of the drugs during the 8 weeks of treatment were 9.9 mg, 3.7 mg, and 5.4 mg, respectively.

A written informed consent was obtained from all patients and proxies. The study was approved by the ethics committee of Lund University, Sweden, and was carried out in accordance with the Helsinki Declaration.

### MMSE and AQT

Specialized dementia nurses administered both tests according to standardized guidelines to maximize interrater reliability. The attention part of the MMSE was scored by the serial subtraction of 7 from 100 [20]. The backward spelling was used if the patient could not perform simple arithmetic exercises [21].

AQT measures attention and cognitive speed, has shown high test-retest reliability (*r *= 0.91 to 0.95), and exhibits no habituation or learning in repeated trials over 10 minutes [22]. AQT-CF has been validated against WAIS-III P IQ (*r *= -0.61; *P *< 0.01), MMSE (*r *= -0.72; *P *< 0.01) and ADAS-cog (*r *= 0.63; *P *< 0.01; correlation made after 6 months of ChEI treatment in AD) [12,23]. It has shown no significant correlation with the Trail Making Test (TMT), verbal association fluency (FAS), or Rey Complex Figure Test (RCFT) [12]. The test scores constitute the number of seconds it takes for a patient to complete each test plate (Figure 1). The test was performed in Swedish, which has produced the same results as a test performed in English [10]. Only AQT-CF was analyzed in this study because it is the most validated and sensitive part of AQT and contains the cognitive measures that are mostly associated with AD [22].

### Assessing test changes: Reliable Change Index

RCI provides a confidence interval (CI), which represents the predicted changes that would occur if a patient's test score does not change significantly from one assessment to another. The most commonly used CI is 90% [24-27], which was also used in this study. With this CI, about 5% in a stable control group will show a test improvement (according to a cut-off value based on the RCI), even when no intervention or real change has occurred. The RCI is calculated from a control group by considering the test-retest reliability, SD, and a systematic bias of the score change between the first and second test occasion (for example, training effect or disease progression) [24]. The formulae that describe this can be found in Additional file 1. Instead of calculating the RCI based on changes in a healthy control group, the RCI was calculated from the changes of the AD patients during the untreated period. The MMSE and AQT changes from before baseline to baseline were thus used to calculate an interval of "normal" test changes when no treatment was given (that is, the RCI). Test changes during the treatment period greater than the RCI were considered to be due to treatment effect. By using the same population as controls (the period from before baseline to baseline) and as cases (the period from baseline to postbaseline), one eliminates many confounding factors such as test-score variability (which is more pronounced in AD than in healthy controls), age, disease progression, gender, and so on.

Because of the clinical nature of this study, the test interval before treatment varied from 1 to 6 months, with a mean ± SD interval of 3.7 ± 1.2 months. Because of the progressive nature of AD, a longer prebaseline test interval would likely show a greater deterioration. Therefore, an approximated score at 8 weeks before baseline was calculated for each patient. The 8 weeks prebaseline score was calculated in the following way: 8 × (baseline score - prebaseline score)/Number of weeks between the prebaseline and baseline visit. These approximated scores were then used to calculate the test changes during 8 weeks before treatment (baseline score - 8 weeks prebaseline score), which provided a single test-retest interval to be compared with the changes after 8 weeks of treatment.

For patients with a test interval of 1 to 3 months before baseline, the mean 8 weeks prebaseline MMSE score was 22.7 ± 3.3 points, and the mean 8 weeks prebaseline AQT-CF score was 97.3 ± 22.8 seconds. Those with an interval of 4 to 6 months before baseline had a mean 8 weeks prebaseline MMSE score of 23.1 ± 3.1 points and a mean prebaseline AQT-CF score of 99.4 ± 21.4 seconds. No significant differences were found between the groups regarding the calculated 8 weeks prebaseline MMSE and AQT scores (*P *> 0.50). Consequently, the fact that AQT and the MMSE were administered at different intervals before treatment did not seem to have any impact on the calculated 8 weeks prebaseline scores. Previous RCI studies have also used a varied interval between test occasions, but without correcting for this (calculating a single test-retest interval) or testing the homogeneity of the group [24,26,27]. We believe our method provides a more valid RCI result because the calculations are based on the same interval (8 weeks without treatment) to which it is going to be applied (8 weeks with treatment).

### Statistical analysis

The RCI was calculated as described in previous studies (see Additional file 1) [27]. Variables that followed a normal distribution were analyzed with parametric statistics, and significantly skewed variables, with nonparametric statistics. The MMSE and AQT changes were assessed with the Wilcoxon matched-pairs signed ranks test. The test changes expressed as percentages were analyzed with the paired *t *test. The McNemar test was used when comparing the number of MMSE and AQT responders. Linear relations were examined by using Pearson correlation. The statistical analyses were performed by using Statistical Package for Social Sciences (SPSS) software (version 17.0; SPSS Inc., Chicago, IL).

## Results

### Changes in test scores

The MMSE and AQT scores are shown in Table 1. It is important to note that a negative AQT change and a positive MMSE change stand for improvement. During the 8-week pre-baseline period when the patients had not yet received any treatment, mean AQT deteriorated significantly by 2.6 seconds (*P *< 0.05), whereas the mean MMSE deteriorated nonsignificantly by -0.29 points (*P *= 0.09). After 8 weeks of treatment, the mean AQT score improved by -9.7 seconds compared with baseline (*P *< 0.0001), and the mean MMSE score improved 0.6 points (*P *< 0.05; Table 1).

**Table 1:** Mean MMSE and AQT values ± standard deviation

Variable	**8 weeks before baseline**^**e**^	Baseline	8 weeks after baseline
	*n *= 75	*n *= 75	*n *= 75
The MMSE, points	23.0 ± 3.1	22.7 ± 3.0^a^	23.3 ± 3.5^b*^
AQT Color-Form, seconds	98.8 ± 21.6	101.5 ± 24.9^c*^	91.8 ± 28.4^d*^

^a^*P *= 0.088 compared with 8 weeks before baseline. ^b^*P *= 0.047 compared with baseline. ^c^*P *= 0.045 compared with 8 weeks before baseline. ^d^*P *< 0.0001 compared with baseline. ^e^Calculated value for all 75 patients according the description in Materials and methods. *Significant change.

Calculated with the Wilcoxon test. MMSE, the Mini-Mental State Examination (0 to 30 points); a higher score indicates better cognition. AQT, A Quick Test of cognitive speed (measured in seconds); less time indicates better cognition.

**Figure 2:**
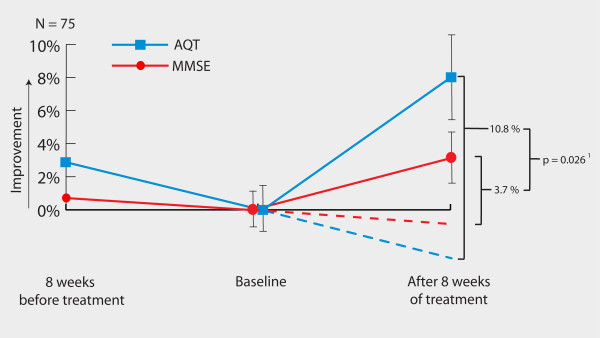
**Mean values of the score changes expressed as percentages**. The lines show the changes from 8 weeks before baseline to baseline and from baseline to 8 weeks after baseline. Dashed lines represent assumed deterioration without treatment. Error bars represent standard error of the mean. ^**1 **^Comparison of the AQT and MMSE improvements after treatment when accounting for disease progression (calculated with paired samples *t *test).

To compare the test changes of MMSE and AQT in a statistical manner, the score changes of each patient must be expressed as a percentage of the previous score because the tests consist of different scales (Figure 2). When just comparing the AQT and MMSE changes *after *treatment, AQT indicated a somewhat more pronounced improvement than did the MMSE (*P *= 0.06). However, it is important to account for the individual disease-progression rate (score change before treatment) because this affects the degree of change in test scores after treatment. The individual test changes of AQT and MMSE during the 8 weeks before treatment were thus subtracted from the changes after 8 weeks of treatment. This meant that if a patient deteriorated 5% in a test score before treatment and improved 10% after treatment, the total treatment effect was an improvement of 15% (assuming that the patient would continue to deteriorate 5% during the 8 weeks after baseline if no treatment had been given). After correcting for individual disease progression, AQT improved by 10.8%, and the MMSE improved by 3.7% (Figure 2) When analyzing these values, the improvement of AQT was significantly greater than that of the MMSE (95% CI of the difference: 0.9% to 13.3%; *P *= 0.026).

### Treatment responders according to the Reliable Change Index

**Figure 3:**
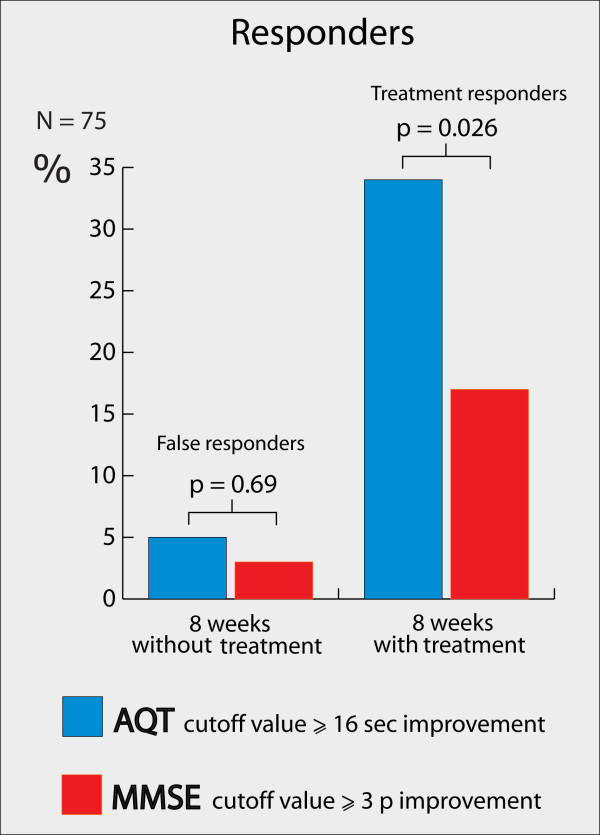
**Responders**. Percentage responders after 8 weeks without treatment and after 8 weeks with treatment according to cut-offs derived from RCI. Details on the RCI analysis can be found in Additional file 1. Calculated with the McNemar test.

The Reliable Change Index (RCI) results are summarized in Figure 3. The test-retest reliability (Pearson correlation) used in the RCI formula was based on the baseline and 8-week pre-baseline occasions. The correlation coefficient of AQT was 0.87 (*P *< 0.001), and for the MMSE, 0.86 (*P *< 0.001). The 90% CI to state if a significant change had occurred on an individual basis (the RCI) was -15.5 to +20.5 seconds for AQT. That is, if a patient improved more than -15.5 seconds, a significant improvement had occurred (clinically this meant that everyone with a -16-second improvement, because only whole seconds were measured). Patients who improved significantly were denoted "responders". For the MMSE, the RCI was -2.99 to +2.41 points (that is, those with at least a +3-point improvement were responders). After 8 weeks of treatment, AQT detected 26 treatment responders (34%), whereas the MMSE detected 13 (17%) treatment responders (Figure 3). As expected according to the RCI, both test cut-offs falsely classified ≤5% responders during the pretreatment period (Figure 3). A "false responder" in this case is a patient who improved more than the RCI during the period when no treatment was given. After treatment, ≤5% of all the patients deteriorated more than the RCI of AQT and the MMSE, which also is just as expected.

Unsurprisingly, the AQT-treatment responders showed greater improvement after 8 weeks of treatment compared with the nonresponders in mean AQT score (*P *< 0.0001). However, a major significant difference in mean AQT change between the groups was still found after 6 months of treatment. The AD patients who were classified as treatment responders by AQT after 8 weeks of treatment showed a mean improvement of -19.3 ± 22.3 seconds on AQT after 6 months of treatment. The nonresponders, conversely, deteriorated 3.3 ± 13.5 seconds over the 6-month treatment period. Thus, the AQT responders at the 8-week visit continued clearly to show a better treatment response at the 6-month visit compared with the nonresponders (*P *< 0.0001).

## Discussion

In this study, we evaluated AQT as a test for detecting early ChEI treatment response in AD and compared it with the MMSE. After 8 weeks of treatment, AQT had improved significantly more than the MMSE when accounting for disease progression (Figure 2). Further, AQT identified twice as many treatment responders as did the MMSE (34% compared with 17%; p = 0.02; Figure 3). The increased number of responders cannot be explained by low reliability or random changes of AQT scores, because AQT classified only 5% (false) responders during the 8-week period before treatment (Figure 3). Finally, when comparing the AQT responders and nonresponders from the 8-week visit, the responders still showed a significantly better treatment response after 6 months of treatment. This indicates that AQT detects early treatment responders who seem to continue to benefit from ChEI treatment.

### Evaluation of treatment

Good clinical practice and cost-benefit considerations require that all AD patients be evaluated before and after the initiation of treatment to determine whether the treatment shall continue [5]. The most common test for this evaluation in clinical practice is the MMSE, and this is also the recommended test according to the NICE guidelines [5]. In clinical trials, the ADAS-cog is the most commonly used cognitive test [28,29]. It measures a broader span of cognitive functions, but has the disadvantage of taking 45 minutes to administer compared with 3 to 5 minutes for AQT and 10 to 15 minutes for the MMSE. Because of the length of the ADAS-cog, it cannot really be regarded as a brief cognitive test suitable for clinical practice. ADAS-cog and the MMSE are well studied for ChEI evaluation of AD, but no previous studies of AQT were performed in this context. However, in a recent randomized, placebo-controlled, multinational study, AQT was used to evaluate the treatment effect of memantine on dementia with Lewy bodies and Parkinson disease dementia [30]. In that study, both AQT and the global cognitive measure CGIC improved significantly after 24 weeks of treatment, compared with placebo, whereas the MMSE failed to improve significantly.

### Future evaluation issues

In the future, it is likely that more patients with mild cognitive impairment (MCI) will be included in therapeutic trials and treated in clinical practice. It is then essential to have a sensitive test with no ceiling effect. The MMSE and the ADAS-cog have detected in MCI studies significant cognitive changes [31,32], but they have also been criticized for their ceiling effects and inability to detect small cognitive changes [28]. In the only study in which AQT has been used to evaluate MCI treatment, AQT improved significantly, whereas the other cognitive tests failed to do so (WAIS III Digit Span, WAIS-R NI Spatial Span, Digit Symbol Modalities, and Rey's Complex Figure Test) [33]. Further, AQT has no ceiling effect and, in this study, was able to significantly detect the subtle disease progress of AD during the nontreatment period of 8 weeks (Table 1). Although the results are promising, more studies are needed to warrant the sensitivity of AQT to cognitive change and to systematically compare it with the MMSE and the ADAS-cog.

Another important future issue is that by 2040, it is predicted that 71% of all dementia patients will be in developing countries [34]. Therefore, the International Psychogeriatric Association (IPA) and the Alzheimer's Association have pointed out the need for a culturally independent test [7,28]. AQT has so far been validated in Western, Arabic, and African countries and does not exhibit any culturally dependent questions or exercises [8,9,35], whereas the MMSE is affected by ethnicity [36,37].

### Detecting treatment responders

In the present study, we evaluated the treatment response after 8 weeks. Previously, it was shown that 4 to 8 weeks of AD treatment results in a significant treatment effect compared with placebo [38-41]. This supports our evaluation of treatment effects already after 8 weeks. It is also is in agreement with the guidelines by NICE and the American College of Physicians (ACP) [5,7]. When evaluating the treatment response, the ACP has suggested that a 3-point change in the MMSE indicates a clinically significant change [7]. This is also the same result as the present study found to indicate a significant change (Figure 3). Unfortunately, no comparable studies are available regarding individual change on AQT.

This study used a statistical method (RCI) to determine treatment responders according to the MMSE and AQT. The clinical relevance of an MMSE improvement of at least 3 points or an AQT improvement of at least 16 seconds is uncertain. In the entire population, the clinical relevance of a mean AQT improvement of 10.8% and a mean MMSE improvement of 3.7% is also uncertain. It is important to note that these values were only used to compare the MMSE and AQT as evaluation instruments. To determine a clinically meaningful AQT or MMSE change, a minimal clinically important difference (MCID) must be defined. One approach to determine the MCID for AD could be to measure the natural history of decline over 12 months or longer in a large group of patients by using AQT, the MMSE, and a global rating of the cognitive performance. A definition of MCID could then be the percentage of change on the MMSE or AQT that is anchored against the natural history of global change in AD.

According to the cut-off values, AQT detected significantly more responders after 8 weeks of treatment than did the MMSE (34% compared with 17%; *P *= 0.026), while falsely detecting 5% responders when no treatment was given (Figure 3). This indicates that AQT is a more-sensitive evaluation tool, which is further emphasized by the changes on a group level. AQT improved significantly more after treatment than did the MMSE when accounting for disease progression (Figure 2). The more-pronounced sensitivity of AQT compared with the MMSE might be explained by their different scales and the different cognitive functions that are measured. Studies have shown that ChEI mostly improves attention [13,14], which is one of the main cognitive domains measured by AQT. It is possible that the treatment response in our study could have been higher if all ChEI doses had been increased after 4 weeks of treatment (the dose was often increased after 8 weeks). This should, however, not affect the comparison between AQT and the MMSE.

Intuitively, it seems that patients who exhibit the right characteristics initially to have a positive treatment response would continue to benefit from the medication. This assumption has been debated, and to determine whether it is true, the reliability and validity of the evaluation instrument must be high. In our study, we found that the AD patients who were classified as treatment responders by AQT after 8 weeks of treatment still performed significantly better on AQT after 6 months, compared with the patients classified as nonresponders after 8 weeks (22.6 seconds in mean difference; *P *< 0.0001). This indicates that AQT might be used after 8 weeks of ChEI treatment to identify those who will continue to benefit from the treatment.

Two advantages of this study are that the treatment was evaluated prospectively and that the same population was used both as controls and as cases. The latter is the most important factor for reliable RCI results, as most confounding factors are eliminated. A shortcoming was that this was not a randomized study with a placebo group, but instead a study with a control group. The treatment effect can therefore not with certainty be separated from the placebo effect. However, placebo treatment in clinical trials of AD patients has not resulted in significant improvements of any cognitive tests [38-41]. Furthermore, the lack of a placebo group should not affect the comparison of the MMSE and AQT.

## Conclusions

In conclusion, AQT, a quick test of cognitive speed and attention, seems to be twice as sensitive as the MMSE in detecting early treatment response to ChEI in AD patients. The early responders detected by AQT continued to benefit from ChEI after 6 months of treatment. This indicates the potential usefulness of AQT when evaluating treatment effects in clinical routine, especially in primary care units. Moreover AQT may be important when evaluating new treatments in the early stages of AD, because of its sensitivity and lack of ceiling effect. Further studies are needed to compare the treatment response detected by AQT and brief cognitive tests other than the MMSE.

## Abbreviations

ACP: American College of Physicians; AD: Alzheimer's disease; AQT: A Quick Test of cognitive speed; AQT-C: A Quick Test of cognitive speed-Color (subtest 1); AQT-CF: A Quick Test of cognitive speed-Color Form (subtest 3); AQT-F: A Quick Test of cognitive speed-Form (subtest 2); ChEI: cholinesterase inhibitors; CI: confidence interval; MMSE: the Mini-Mental State Examination; NICE: National Institute for Health and Clinical Excellence; NINCD-ADRDA: National Institute of Neurological and Communicative Disorders and Stroke and the Alzheimer's Disease and Related Disorders Association; *r*: correlation coefficient; RCI: Reliable Change Index; SD: standard deviation.

## Competing interests

The authors declare that they have no competing interests.

## Authors' contributions

SP participated in the design of the study, performed the statistical analysis, and drafted the manuscript. LM and EL participated in the design and coordination of the study and revised the manuscript. CW revised the statistical analysis and the manuscript. OH participated in the design of the study, helped out in the statistical analysis, and revised the manuscript. All authors read and approved the final manuscript.

## Supplementary Material

Additional file 1 **Reliable Change Index (RCI)**. Statistical information on how the RCI was calculated. Click here for file
