# Supplementary material for: A Quick Test of cognitive speed is sensitive in detecting early treatment response in Alzheimer's disease
Source: Alzheimers Res Ther. 2010 Oct 15;2(5):29. doi: 10.1186/alzrt53 (PMC2983438; doi:10.1186/alzrt53)
Supplement: Additional file 1 — Reliable Change Index (RCI). Statistical information on how the RCI was calculated. [file alzrt53-S1.DOC]

**Additional file 1**

Reliable Change Index (RCI)

The RCI was calculated by first establishing the standard error of measurement (SE_meas_ ), which corrects for possible measurement errors:

$$SE_{meas}=SD\sqrt{1-r}$$

*SD = Standard deviation of the test score at 8 week pre-baseline
r = The test-retest reliability coefficient (Pearson correlation) based on the 8 weeks pre-baseline and baseline scores*

AQT-color form (AQT-CF):

$$SE_{meas}=21.63\sqrt{1-0.870}=7.80$$

MMSE:

$$SE_{meas}=3.12\sqrt{1-0.859}=1.17$$

From the SE_meas_ the standard error of differentiation was calculated (SE_diff_):

$$SE_{diff}=\sqrt{2*(SE_{meas})^{2}}$$

The SE_diff_ was then used to create a 90 % CI (the RCI).

$$RCI=\pm SE_{diff}*1.64$$

RCI for AQT:

$$RCI=\pm11.03*1.64=\pm18.1\sec$$

RCI for MMSE:

$$RCI=\pm1.66*1.64=\pm2.7p$$

To account for the disease progression, the change in mean score from 8 weeks pre-baseline to baseline was subtracted from the RCI. To determine if a patient had made a significant improvement after treatment, the following had to be fulfilled:

*A patient’s test change after treatment > RCI - X_diff_*

*X_diff_ = Mean test score at pre-baseline subtracted by the mean test score at baseline*

*X_diff_ AQT-CF = -2.6 sec*

*X_diff_ MMSE = 0.29 p*

When correcting for disease progression the RCI for AQT-CF was

*-18.1 – -2.6 to +18.1 – -2.6* = *-15.5 sec to 20.5 sec*

And the corrected RCI for MMSE was

*-2.7 – 0.29 to +2.7 – 0.29 = -2.99 p to 2.41 p*

How the RCI was applied to detect treatment responders is described in “Results” – *“Treatment Responders According to the RCI”.* The number of treatment responders according to the RCI analysis is presented in Figure 3.
